# Acute Allergic Reactions and Severe Anaphylaxis: Underlying Causes, Management Strategies, and Future Directions

**Published:** 2026-04-06

**Authors:** Aris Ellorin, Devendra K. Agrawal

**Affiliations:** 1 Department of Translational Research, College of Osteopathic Medicine of the Pacific, Western University of Health Sciences, Pomona, California 91766, USA

**Keywords:** Allergic diseases, Anaphylaxis, Emergency medicine, Epinephrine, IgE, Immune tolerance, Immunotherapy, Mast cells, MRGPRX2, Platelet-Activating Factor

## Abstract

Anaphylaxis represents the most severe and potentially fatal manifestation of allergic disease, characterized by sudden multi-system involvement and rapid hemodynamic compromise. While management protocols have improved, global incidence continues to rise and preventable deaths persist — driven largely by delayed epinephrine administration and inadequate long-term follow-up. This narrative review synthesizes current evidence on the immunological and non-immunological mechanisms underlying anaphylaxis, evaluates diagnostic criteria from major allergy societies, and appraises both immediate and long-term treatment strategies. Contributing factors to persistent morbidity — including epinephrine underuse, barriers to autoinjector access, and the emergence of biphasic and refractory phenotypes — are examined in depth. Advances in novel epinephrine delivery platforms, biologic therapies targeting the IgE and cytokine axes, and immunomodulatory strategies including oral and venom immunotherapy are highlighted as promising avenues for improving outcomes. This review also underscores the need for validated predictive biomarkers, equitable device access, and prospective trials to close the gaps that continue to drive preventable mortality.

## 1. Introduction

Allergic diseases affect approximately up to 30% of the global population and impose a substantial public health burden [1–8]. Among them, anaphylaxis is the most severe manifestation, characterized by acute multi-system involvement and rapid hemodynamic deterioration [9–12]. The case-fatality rate, while low at 0.25–0.33% of hospitalizations, translates to 63–99 deaths annually in the United States alone [13]. A recent systematic review reported a 7.4% annual increase in all-cause anaphylaxis incidence globally between 1990 and 2017, with pediatric rates rising disproportionately in suburban compared to urban settings [14,15].

Timely intramuscular epinephrine remains the only intervention consistently shown to reduce morbidity and mortality, yet community underuse is widespread — documented in up to 64% of pre-hospital events [16,17]. Contributing factors include poor autoinjector carriage, inadequate patient and provider training, socioeconomic barriers, and fear of adverse effects [16–19]. The emergence of biphasic and refractory anaphylaxis phenotypes further complicates management, as recurrent or epinephrine-resistant reactions carry substantially higher morbidity [20].

Recent advances offer meaningful clinical progress. Next-generation intranasal epinephrine formulations and high-dose autoinjectors address delivery barriers, while biologics targeting IgE and cytokine axes and allergen immunotherapies inducing sustained tolerance expand long-term options [11, 21–23]. Precision biomarkers such as hereditary α-tryptasemia, platelet-activating factor (PAF) acetylhydrolase deficiency, and elevated baseline serum tryptase, and abnormal density and function of immune cell functions have emerged as candidates for improved individual risk stratification [24–37]. This review highlights these developments and outlines a research agenda for areas where critical gaps remain.

## 2. Epidemiology and Disease Burden

### 2.1 Prevalence and Incidence

The lifetime prevalence of anaphylaxis is estimated between 1.6% and 5.1% [16,38]. Global incidence varies substantially by geography and methodology: population-based studies report rates from approximately 26 per 100,000 person-years in South Korea to 82.5 per 100,000 in Western Australia [39,40]. A recent systematic review and meta-analysis by Pühringer and colleagues confirmed a 7.4% annual increase in all-cause anaphylaxis incidence worldwide from 1990 to 2017, with food-triggered cases in pediatric populations demonstrating particularly steep temporal trends [14,41]. Perioperative anaphylaxis, though less common, carries a mortality rate of up to 4.8% and occurs in approximately 100 per million procedures [42].

### 2.2 Underuse of Epinephrine

A persistent and alarming gap exists between the severity of anaphylaxis and real-world epinephrine administration. Only 21% of children and 7% of adults experiencing anaphylaxis in community settings receive epinephrine prior to hospital arrival [43]. Underuse is similarly documented within emergency departments, where guideline‑concordant first‑line epinephrine administration remains inconsistent [44]. This disparity reflects multiple intersecting barriers: cost and limited availability of autoinjectors, patient and caregiver anxiety regarding injection, misconceptions about contraindications, and insufficient training in device use [18,19,44]. Addressing these barriers is a public health priority, as pre-hospital epinephrine administration is consistently associated with reduced biphasic risk and shorter emergency department stays [20,45].

## 3. Diagnostic Criteria and Clinical Definitions

Consensus definitions from the World Allergy Organization (WAO) and NIAID/FAAN require the acute onset of characteristic symptoms involving at least two organ systems following exposure to a likely allergen, or isolated hypotension after a known trigger [38]. The 2020 WAO guidance refined these criteria to better differentiate anaphylaxis from isolated cutaneous or respiratory reactions [38]. This update also emphasized recognition of atypical presentations, particularly in infants, older adults, and perioperative settings [38]. Ongoing discussions about further revision include whether and how to incorporate cardiovascular‑specific entities such as Kounis syndrome, in which coronary artery spasm or acute myocardial infarction is precipitated by the anaphylactic cascade [46].

Diagnosing anaphylaxis remains clinically challenging, as evidenced by the subtle but meaningful differences in criteria across major authoritative bodies (see Table 1) [38]. These discrepancies complicate epidemiological comparisons and contribute to under-recognition in atypical presentations. Ongoing efforts to harmonize definitions internationally will be essential for advancing both research reproducibility and consistent clinical practice [12,38].

## 4. Pathophysiology and Molecular Mechanisms

### 4.1 IgE-Mediated Anaphylaxis

Anaphylaxis is primarily driven by an immunoglobulin E (IgE)-mediated mechanism in previously sensitized individuals [47]. Upon re-exposure to a causative allergen, cross-linking of allergen-specific IgE bound to high-affinity FcεRI receptors on mast cells and basophils triggers rapid degranulation [47]. This releases histamine, tryptase, and proteoglycans into the systemic circulation [47–49].

Downstream signaling cascades subsequently generate prostaglandins, leukotrienes, and platelet-activating factor (PAF), which collectively drive bronchoconstriction, vascular leak, and amplified systemic inflammation [47–49]. Elevated PAF levels have been associated with greater anaphylaxis severity [26]. Effectors cells are recruited by TNF-α resulting in the sustenance of multi-organ symptoms including urticaria, angioedema, bronchospasm, hypotension, and gastrointestinal distress [47]. This cascade helps explain why antihistamines alone are inadequate and why epinephrine — acting across multiple receptor classes simultaneously — remains the cornerstone first‑line intervention [49].

### 4.2 Non-IgE Mediated Pathways and Emerging Mechanisms

While IgE-mediated anaphylaxis is the most prevalent form, the reaction can also occur through several IgE-independent mechanisms, including direct mast cell activation, immune complex–driven complement activation, cytotoxic reactions, neuropeptide release, and T-cell–mediated pathways [50]. Research using murine models has illuminated the role of IgG antibodies and FcγR-expressing effector cells — including basophils, macrophages, and neutrophils — in triggering PAF-mediated anaphylaxis [50]. However, the exact significance of IgG-mediated mechanisms in human anaphylaxis remains incompletely defined.

At the molecular level, tyrosine kinases including Lyn, Syk, and Fyn modulate FcεRI signal transduction, and their dysregulation may contribute to exaggerated mast cell responses [48]. Activating mutations such as the D816V variant in the KIT tyrosine kinase — frequently observed in systemic mastocytosis — predispose affected individuals to recurrent, unexplained anaphylaxis through constitutive mast cell activation [24]. Emerging mediators including sphingosine-1-phosphate and nitric oxide are also gaining recognition for their roles in anaphylactic physiology [50]. Separately, the MAS-related G protein-coupled receptor member X2 (MRGPRX2) has been identified as a non-IgE target through which neuromuscular blocking agents and other drugs directly activate mast cells, providing a mechanistic basis for drug-induced anaphylaxis that bypasses the classical IgE sensitization requirement [50]. This evolving mechanistic landscape suggests that future therapeutic strategies and diagnostic biomarker panels may increasingly be individualized to the predominant pathway driving each patient’s reaction.

## 5. Triggers and Risk Amplifiers

Foods constitute the predominant trigger of anaphylaxis in pediatric patients, with peanuts, tree nuts, shellfish, and wheat accounting for most cases [51]. In adults, drug reactions — particularly to β-lactam antibiotics, NSAIDs, and radiocontrast media — are leading causes, with biologics representing an increasingly important emerging category [52]. Venom-induced anaphylaxis from Hymenoptera stings contributes disproportionately to anaphylaxis fatalities across all age groups [53].

Several comorbidities and pharmacological exposures substantially amplify reaction severity. Asthma, cardiovascular disease, and obesity increase the risk of fatal outcomes [12]. Concurrent β-blocker or ACE-inhibitor therapy blunts the physiological response to epinephrine and worsens refractory presentations [12]. Clonal mast-cell disorders — including systemic mastocytosis and hereditary α-tryptasemia — represent the most biologically distinct risk-amplifying conditions, predisposing affected individuals to unprovoked or disproportionately severe reactions through constitutive mediator release [24,54].

## 6. Clinical Phenotypes

Anaphylaxis manifests across three clinically distinct phenotypes with important management implications. Uniphasic reactions, the most common, resolve completely with treatment and do not recur [20]. Biphasic anaphylaxis involves recurrence of symptoms 1–48 hours after apparent resolution [20]. Reported incidence ranges from 4% to 20%, with large emergency department cohorts estimating the rate at approximately 7–16% [20,55,56]. Independent predictors of biphasic recurrence include delayed initial epinephrine administration of more than 60 minutes from onset, drug or idiopathic triggers, and angioedema at presentation [20]. Supporting this, a nationwide cohort study from U.S. emergency departments found that patients who received prehospital epinephrine had significantly lower odds of biphasic reactions and shorter emergency department stays, reinforcing early intervention as both therapeutic and preventive [45].

Refractory anaphylaxis is defined by persistent or progressive symptoms despite at least two appropriately dosed intramuscular epinephrine injections [20]. This phenotype is associated with delayed presentation, β-blocker use, obesity, and severe initial manifestations including cardiovascular collapse [12,57]. Cases meeting this threshold require escalation to intravenous epinephrine infusion, vasopressors, and advanced resuscitation measures [12,57]. Data from the European Anaphylaxis Registry indicate that refractory cases are underreported and that standardized recognition and management protocols remain inconsistently applied across institutions [57].

## 7. Immediate Management

**Figure 1:**
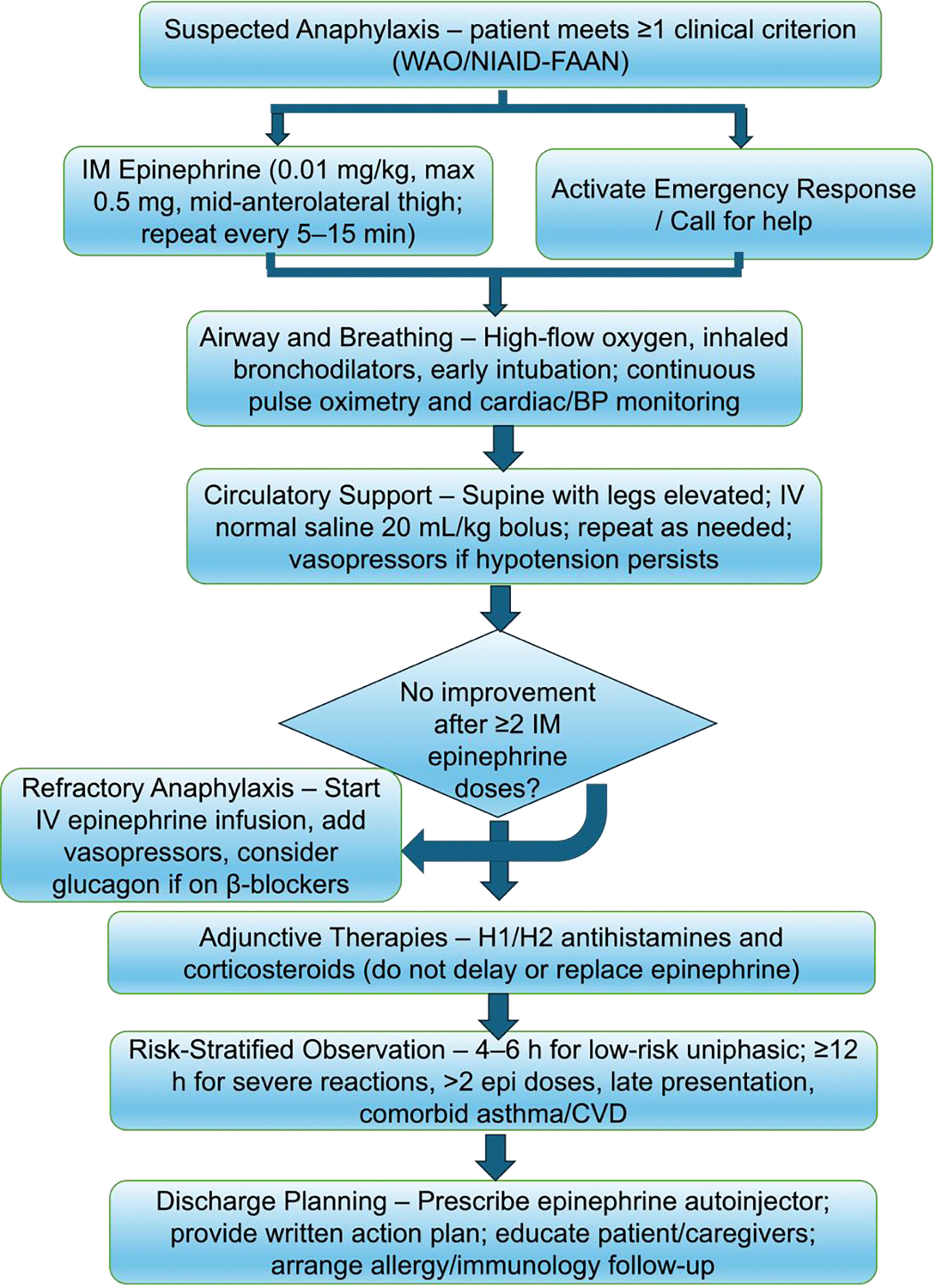
Flow chart illustrating recommended steps for the acute management of anaphylaxis, adapted from current WAO and emergency medicine practice guidelines. Key interventions include prompt intramuscular epinephrine, airway support (high-flow oxygen, bronchodilators, intubation as indicated), circulatory stabilization (supine positioning, isotonic fluid bolus, vasopressors for refractory hypotension), adjunctive antihistamines and corticosteroids, and risk-stratified observation periods [12,38,60].

The cornerstone of acute anaphylaxis management is prompt intramuscular epinephrine [12]. Delivered at 0.01 mg/kg (maximum 0.5 mg) into the mid-anterolateral thigh, epinephrine simultaneously reverses bronchoconstriction, restores vascular tone, and suppresses further mediator release [12]. Early administration is associated with reduced biphasic risk and shorter emergency department observation time [20]. Despite this evidence, epinephrine remains underused in both community and hospital settings [16,18]. Provider anxiety, cost barriers, and persistence of misconceptions about side effects have been identified as modifiable targets for intervention [16,19,44] (Figure 1).

Airway management proceeds in parallel with epinephrine administration. High-flow supplemental oxygen should be initiated immediately [12]. Patients with bronchospasm benefit from inhaled bronchodilators, while those with impending airway compromise require early intubation [12]. Ketamine is a preferred induction agent in this context given its bronchodilatory properties and hemodynamic stability profile [58–60]. Circulatory support requires supine positioning with leg elevation to optimize venous return, followed by isotonic crystalloid boluses of 20 mL/kg for persistent hypotension after epinephrine, with vasopressor escalation if cardiovascular collapse remains refractory [12,60] (Figure 1).

Adjunctive agents — including H1 and H2 antihistamines and corticosteroids — address cutaneous symptoms such as urticaria and may reduce late-phase inflammatory activity, but neither class prevents biphasic reactions or substitutes for epinephrine [12,16]. Observation following clinical stabilization is essential to detect symptom recurrence. Evidence-based protocols support 4–6 hours of monitoring for low-risk uniphasic presentations [12,16]. Extended observation of at least 12 hours is recommended for severe initial reactions, delayed epinephrine administration, or cases with significant comorbidities [12,16].

## 8. Long-Term Management and Secondary Prevention

Long-term anaphylaxis management requires a personalized, multi-pronged strategy centered on trigger avoidance, immunotherapy, and structured education. Allergen avoidance remains the most reliably effective prevention strategy [12,61–63]. Accurate trigger identification, often requiring referral to an allergist for skin-prick testing, specific IgE measurement, and controlled challenges, is foundational to developing tailored avoidance plans [12,63].

Allergen-specific immunotherapy (AIT) offers the most durable long-term risk reduction for select populations. Venom immunotherapy (VIT) provides a 90% reduction in the risk of future anaphylaxis from Hymenoptera stings and is considered standard of care for patients with a documented history of venom-triggered systemic reactions [53]. Oral immunotherapy (OIT) has expanded options for patients with peanut and other food allergies, enabling clinically meaningful desensitization through carefully titrated exposure protocols [23]. However, adherence to daily maintenance dosing is critical, and clinicians must monitor closely for treatment-emergent eosinophilic esophagitis [23].

Biologic therapies have emerged as an important adjunct for patients with severe, recurrent, or refractory allergic disease. Omalizumab, a monoclonal anti-IgE antibody, reduces free IgE levels and raises the allergen threshold required to trigger mast cell activation [64,65]. Dupilumab and anti-IL-5 agents similarly modulate the Th2-driven inflammatory milieu underlying allergic sensitization [43]. Reviews of clinical trial data suggest that these agents can meaningfully reduce reaction frequency and corticosteroid burden during allergen immunotherapy [43]. However, optimal patient selection, treatment duration, and combination sequencing with AIT remain active areas of investigation [64].

Novel epinephrine delivery platforms represent a meaningful advance for device adherence and patient acceptability. Intranasal epinephrine formulations have demonstrated pharmacokinetic profiles comparable to standard autoinjectors in clinical studies [21]. High dose autoinjectors provide more rapid peak plasma concentrations in patients for whom standard dosing may be inadequate [22]. Patient and caregiver preference data suggest that many individuals favor needle‑free delivery methods, indicating that these platforms may help reduce some behavioral barriers to pre‑hospital epinephrine use. [21,22].

Education and structured emergency action plans constitute the behavioral infrastructure of secondary prevention. Comprehensive training programs increase autoinjector carriage and correct technique, and community‑level educational initiatives have been associated with improved preparedness and guideline‑concordant management in community anaphylaxis [63].

Advances in pharmacogenomics and biomarker science are helping to individualize care. Genetic screening for hereditary α-tryptasemia identifies patients at higher risk for severe reactions [24]. The KIT D816V mutation can guide decisions about the need for prolonged hospital observation and personalized monitoring strategies in mastocytosis-related anaphylaxis [24]. Dynamic biomarkers including serial tryptase levels and PAF concentrations further refine individual risk assessment in the acute setting [25,26].

## 9. Discussion

This review addresses a clinically relevant and evolving topic: the persistent gap between the availability of effective treatments and their real-world uptake [16–18]. Several prior reviews have addressed anaphylaxis mechanisms or management in isolation, but the present synthesis integrates pathophysiology, phenotypic classification, emerging therapeutics, and patient-level barriers within a unified framework [48,50].

A particularly actionable implication concerns the consistent failure to administer epinephrine promptly. Pre-hospital administration remains below 40% in most community settings, and within emergency departments, guideline-concordant first-line epinephrine use is inconsistently documented [16,17]. This is not exclusively a knowledge failure — it reflects structural gaps in autoinjector accessibility, prescribing rates, and follow-up education [19]. Policy interventions modeled on public access defibrillation programs may offer a scalable template for increasing community-level epinephrine availability [19].

The mechanistic findings reviewed here also suggest important precision medicine opportunities. The identification of MRGPRX2-mediated mast cell activation in drug-induced anaphylaxis and the role of KIT mutations in mastocytosis-related anaphylaxis collectively indicate that anaphylaxis is not a single immunological entity but a syndromic spectrum [24,50]. As biomarker science advances, diagnostic panels encompassing PAF levels, tryptase kinetics, and genetic variants may enable individualized risk stratification that goes beyond current binary treatment algorithms [25,26].

A notable limitation of this review is the variable quality and heterogeneity of underlying studies [20,55,56]. Many key estimates — including biphasic reaction rates and epinephrine underuse figures — derive from retrospective, single-center, or regionally limited cohorts [20,55,56]. Prospective, multi-site, population-representative studies are needed to generate estimates robust enough to inform guideline revision [12,14].

## 10. Conclusion

Anaphylaxis management has advanced considerably, yet the burden of preventable deaths remains unacceptably high [13,16]. Bridging the gap between current evidence and clinical practice demands simultaneous attention to biomedical, behavioral, and systemic dimensions of care [12,20]. The field requires prospective validation of biphasic and refractory risk prediction tools, large multi-center cohort studies to validate dynamic biomarker panels for bedside use, and rigorous randomized trials to define optimal sequences of biologic and immunotherapeutic interventions [23–26,65]. Equally important are policy-level efforts to ensure equitable autoinjector access and the integration of structured emergency action plans into every patienťs discharge planning [19,63]. Addressing these priorities collectively represents the most direct path to improving outcomes for patients with severe allergic disease worldwide.

## Figures and Tables

**Table 1:** Anaphylaxis criteria by guidelines: Side-by-side comparison of consensus criteria for the clinical diagnosis of anaphylaxis from the World Allergy Organization (WAO), NIAID/FAAN, and other leading societies. The diagram emphasizes clinically relevant differences in organ system thresholds, recognition of atypical presentations, and conditional hypotension criteria. This comparison illustrates the ongoing need for harmonized definitions to support epidemiological research and consistent clinical practice [21,38,46].

Criterion	NIAID/FAAN 2006 Guidelines	WAO 2020 Guidelines
Clinical scenario 1	Acute onset (minutes-hours) with skin/mucosal involvement (e.g., hives, flushing, swollen lips/tongue) and either respiratory compromise or reduced BP/end-organ dysfunction.	Acute onset with typical skin symptoms (urticaria, flushing, angioedema) and significant symptoms from at least one other organ system (respiratory, cardiovascular, or severe GI).
Clinical scenario 2	Two or more of the following occurring rapidly after exposure to a likely allergen: skin/mucosa, respiratory compromise, reduced BP, or persistent GI symptoms.	Two or more of respiratory compromise, reduced BP, or severe GI symptoms after exposure to a likely allergen, even in the absence of skin involvement.
Clinical scenario 3	Reduced BP after exposure to a known allergen for that patient.	Reduced BP after exposure to a known allergen, defined as a >30% decrease from baseline or age-specific hypotension.
Skin Involvement	Required for scenario 1; common in scenario 2.	Not required; diagnosis can be made without skin findings in high-risk scenarios (e.g., perioperative).
GI Involvement	Included as one of the systems in scenario 2.	Emphasized as potentially severe and sufficient as part of multi-system involvement.
Key Clinical Differences	More skin-centric; less explicit guidance for atypical or cardiovascular-dominant presentations.	Allows anaphylaxis without skin signs; clearer hypotension thresholds; improved recognition of atypical and perioperative forms.

## Key Points

- Anaphylaxis incidence increases approximately 7.4% annually, with pediatric rates rising disproportionately in suburban compared to urban settings
- Pre-hospital epinephrine is consistently associated with reduced biphasic risk and shorter emergency department stays, yet underuse persists in up to two-thirds of community events
- Delayed epinephrine (>60 min), drug or idiopathic triggers, and angioedema are the strongest independent predictors of biphasic recurrence
- Defined by failure of ≥2 intramuscular doses, refractory anaphylaxis requires urgent escalation to intravenous epinephrine and vasopressors
- Venom immunotherapy reduces future sting anaphylaxis risk by 90%; OIT and anti-IgE/IL-4/IL-5 biologics reduce food-allergic reaction frequency and threshold
- Intranasal epinephrine and high-dose autoinjectors show pharmacokinetic profiles comparable to standard devices, with emerging evidence suggesting patient preference for needle-free delivery
- Autoinjector cost, limited public access, and training disparities fuel preventable deaths, particularly in low-resource settings
- Prospective biomarker validation, population-representative epidemiological studies, and rigorous immunotherapy sequencing trials are the most critical unmet needs
